# Evidence-based Shared-Decision-Making Assistant (SDM-assistant) for choosing antipsychotics: protocol of a cluster-randomized trial in hospitalized patients with schizophrenia

**DOI:** 10.1186/s12888-022-04036-5

**Published:** 2022-06-17

**Authors:** Spyridon Siafis, Nicola Bursch, Katharina Müller, Lisa Schmid, Florian Schuster, Jakob Waibel, Tri Huynh, Florian Matthes, Alessandro Rodolico, Peter Brieger, Markus Bühner, Stephan Heres, Stefan Leucht, Johannes Hamann

**Affiliations:** 1 Department of Psychiatry and Psychotherapy, School of Medicine, Technical University of Munich, Munich, Germany; 2 kbo-Isar-Amper-Klinik, Munich, Germany; 3 Schön Klinik Roseneck, Rosenheim, Germany; 4 Department of Informatics, Technical University of Munich, Munich, Germany; 5 Department of Clinical and Experimental Medicine, Institute of Psychiatry, University of Catania, Catania, Italy; 6 Psychological Methodology and Diagnostics, Ludwig Maximilian University, Munich, Germany

**Keywords:** Health service research, SDM, EBM, Decision aid, Patient preference, Inpatient psychiatry, Psychosis, Antipsychotic, Response, Side-effect

## Abstract

**Background:**

Choosing an antipsychotic medication is an important medical decision in the treatment of schizophrenia. This decision requires risk-benefit assessments of antipsychotics, and thus, shared-decision making between physician and patients is strongly encouraged. Although the efficacy and side-effect profiles of antipsychotics are well-established, there is no clear framework for the communication of the evidence between physicians and patients. For this reason, we developed an evidence-based shared-decision making assistant (SDM-assistant) that presents high-quality evidence from network meta-analysis on the efficacy and side-effect profile of antipsychotics and can be used as a basis for shared-decision making between physicians and patients when selecting antipsychotic medications.

**Methods:**

The planned matched-pair cluster-randomised trial will be conducted in acute psychiatric wards (*n* = 14 wards planned) and will include adult inpatients with schizophrenia or schizophrenia-like disorders (*N* = 252 participants planned). On the intervention wards, patients and their treating physicians will use the SDM-assistant, whenever a decision on choosing an antipsychotic is warranted. On the control wards, antipsychotics will be chosen according to treatment-as-usual. The primary outcome will be patients’ perceived involvement in the decision-making during the inpatient stay as measured with the SDM-Q-9. We will also assess therapeutic alliance, symptom severity, side-effects, treatment satisfaction, adherence, quality of life, functioning and rehospitalizations as secondary outcomes. Outcomes could be analysed at discharge and at follow-up after three months from discharge. The analysis will be conducted per-protocol using mixed-effects linear regression models for continuous outcomes and logistic regression models using generalised estimating equations for dichotomous outcomes. Barriers and facilitators in the implementation of the intervention will also be examined using a qualitative content analysis.

**Discussion:**

This is the first trial to examine a decision assistant specifically designed to facilitate shared-decision making for choosing antipsychotic medications, i.e., SDM-assistant, in acutely ill inpatients with schizophrenia. If the intervention can be successfully implemented, SDM-assistant could advance evidence-based medicine in schizophrenia by putting medical evidence on antipsychotics into the context of patient preferences and values. This could subsequently lead to a higher involvement of the patients in decision-making and better therapy decisions.

**Trial registration:**

German Clinical Trials Register (ID: DRKS00027316, registration date 26.01.2022).

**Supplementary Information:**

The online version contains supplementary material available at 10.1186/s12888-022-04036-5.

## Background

A large number of antipsychotics are available for the treatment of schizophrenia with substantial differences in their side-effect profiles and smaller in efficacy [1]. This makes the selection of an antipsychotic for the individual more difficult, given also that several factors should be taken into consideration, such as patient preferences, prior experiences, side-effect profiles, comorbidities, age and sex [2–5]. This “preference-sensitive” decision requires shared-decision-making (SDM) between patients and their treating physicians [4, 6]. In addition, physician decision-making behaviours are often not evidence-based, but influenced by irrational factors [7–9], cognitive biases [10, 11], and lack of statistical knowledge [12]. Furthermore, decisions in the treatment of schizophrenia are perceived by many patients as “paternalistic” or dominated by the physician, without patients really having a choice about which drug to be prescribed [13, 14]. These aspects could lead to less optimised drug selection, poor therapeutic relationship, lower adherence and increased relapse rates [15].

**Fig. 1:**
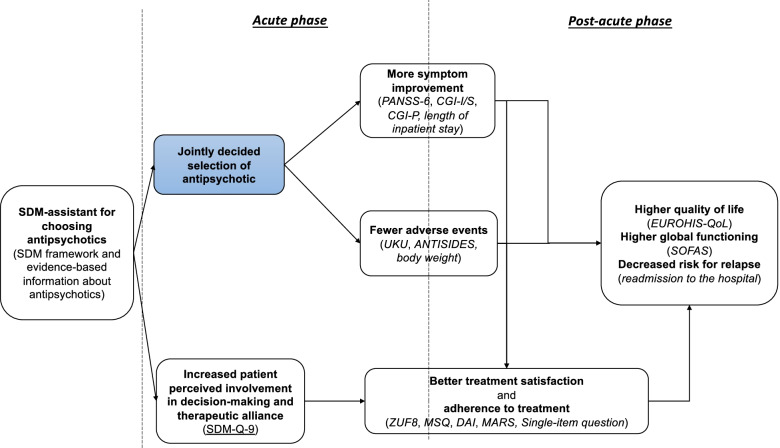
Potential outcomes of SDM-assistant for choosing antipsychotics

In order to overcome the above defects in decision-making, we developed an evidence-based shared-decision-making assistant (SDM-assistant) for choosing antipsychotics. This newly developed tool provides a framework of shared-decision-making illustrating evidence from a network meta-analysis on the efficacy and side-effects of antipsychotics [1]. It may also overcome limitations of previous tools with limited capabilities in shared-decision-making or presenting evidence of lower quality [16]. Patients and practitioners can use the SDM-assistant as a basis of share-decision-making by discussing and navigating differences of antipsychotics. This could facilitate “better” therapy decisions (e.g., fewer adverse events) and increase patient involvement, and thus, could subsequently lead to greater patient satisfaction and, potentially, fewer rehospitalisations due to improved adherence [17, 18]. Figure 1 shows the potential outcomes of using the SDM-assistant for choosing antipsychotics, as adapted by our previous work on shared-decision making in patients with an acute exacerbation of schizophrenia [19].

The planned cluster-randomized-controlled trial aims to compare the effects of SDM-assistant versus treatment-as-usual (TAU) in inpatients with schizophrenia. We will investigate whether the SDM-assistant can improve perceived shared-decision-making, lead to fewer side-effects and better adherence at discharge and three-months after discharge. We will also explore factors that could have an impact on the use and effects of the SDM-assistant tool.

## Methods

### Trial design

The planned study is a cluster-randomized-controlled trial evaluating the use of an evidence-based shared-decision making assistant (SDM-assistant) for choosing antipsychotics in patients with schizophrenia hospitalised in acute psychiatric wards in three hospitals in Munich, Germany (Klinikum rechts der Isar of the Technical University of Munich, Isar-Amper-Klinikum München-Ost and München-Schwabing). The protocol is registered in the German Clinical Trials Register (ID: DRKS00027316, registration date 26.01.2022). The protocol is reported according the SPIRIT [20] and SUNDAE [21] statements.

### Participants

Participants should have a diagnosis of a schizophrenia spectrum disorder (ICD-10 F20-F9) and be hospitalized in an acute psychiatric ward at the time of examination. They should be aged between 18 and 65 years at the time of inclusion and be able to give their informed consent to participate in the study. There are no other exclusion criteria, e.g., in terms of sex, ethnicity, illness stage.

### Intervention and control group

Antipsychotic drugs are recommended for the treatment of acute episodes of schizophrenia [4]. Therefore, decisions on choosing which antipsychotic drug to be used are necessary in acute psychiatric wards. In the intervention group, whenever a decision on choosing an antipsychotic is warranted (e.g., initiation, continuation or change of antipsychotic drugs), patients and their treating physicians will use the SDM-assistant in the context of a shared-decision-making process for the selection of an antipsychotic, in addition to the usual care. The SDM-assistant can be used more than once in different sessions during the trial. In the control group, patients and their treating physicians will not use the SDM-assistant, and antipsychotics will be chosen according to treatment-as-usual (TAU). Changes in the treating physicians will be discouraged, yet, when necessary, they will be recorded. In addition, we will use cluster randomization at the ward level, and therefore, a possible switch to a physician from the other arm of the trial would be unlikely. We will consider as protocol violations when patients do not receive at least one session of the SDM-assistant or when patients switch from the control to the intervention group or vice versa.

### SDM-assistant for choosing antipsychotics

The SDM-assistant is a browser-based-application developed by our team in accordance to the IPDASi v4.0 [22] (eAppendix 1) and provides an interactive framework of shared-decision-making between patients and physicians for the selection of antipsychotics. The SDM-assistant provides a basis for a decision; the final decision of the selection of the drug will be taken in a shared-decision-making process between clinician and patients, and there is no direct treatment recommendation provided by the SDM-assistant tool. Here, reference is made to the three-talk model of SDM, and the work with the decision aid is located in the “option talk” [23]. We included patients and relatives in the development and design of the SDM-assistant, and its final format and way of presenting the data was determined in pre-tests and focus groups.

**Fig. 2:**
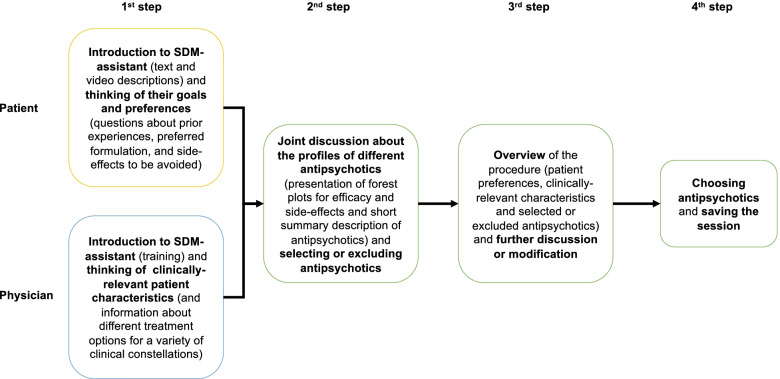
Session of SDM-assistant

Each session of the SDM-assistant is multi-step (Fig. 2). At the very beginning, treating physicians will receive training in the use of the SDM-assistant and will be introduced in the methods of evidence-based-medicine and shared-decision-making. In a first step, users (patients and physicians) work on the SDM-assistant separately. Patients get information from text descriptions and video presentations by health practitioners and ex-users about schizophrenia, aspects of drug treatment with antipsychotics, and shared-decision-making. Patients are then guided to think about their own goals and preferences for the treatment with antipsychotics (e.g., prior experience with antipsychotics, side-effects to avoid, antipsychotic formulation). Physicians, on the other side, enter clinically relevant patient information (e.g., age, sex, duration of illness, comorbidities).

**Fig. 3:**
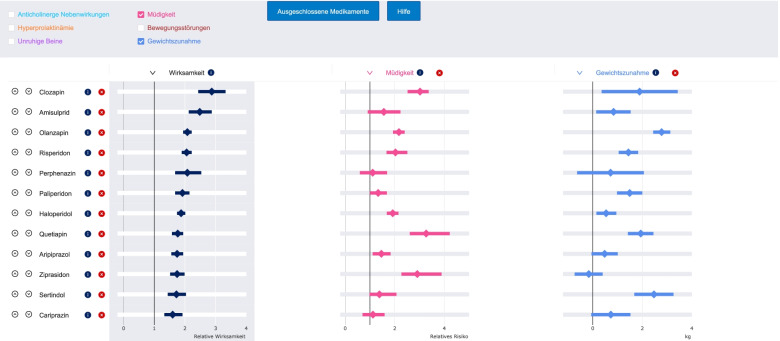
Forest plot interface of the SDM-assistant for choosing antipsychotics. Forest plots presenting effect-sizes (point estimates and their 95% credible intervals) for the comparisons of antipsychotics with placebo. Relative risks are used as the effect-size, except for mean differences in kg for weight gain. The figure was taken as a screenshot from the SDM-assistant for choosing antipsychotics

In a second step, patients and physicians can jointly consult forest plots presenting evidence on antipsychotics (Fig. 3), and subsequently make a shared treatment decision.

In the context of the study, evidence on twelve antipsychotics (i.e., amisulpride, aripiprazole, cariprazine, clozapine, haloperidol, olanzapine, paliperidone, perphenazine, quetiapine, risperidone, sertindole, and ziprasidone) licensed and commonly used in Germany is considered. Other antipsychotics were not considered, e.g. not yet licensed (e.g., brexpiprazole or lurasidone), old and not widely used (e.g., many first-generation antipsychotics) [24] and/or based on limited evidence from RCTs (e.g., fluphenazine or sulpiride) [1]. Published evidence from a large network meta-analysis conducted by our group is used [1]. Network meta-analysis is considered the highest level of evidence in treatment guidelines [25], and thus, can be informative for the shared-decision-making process. Both efficacy and side-effects are important for the selection of an antipsychotic [2, 3], therefore, we consider overall efficacy (reduction of overall symptoms as measured by validated scales) and six important side-effects of antipsychotics, i.e., weight gain, use of antiparkinsonian medications as a proxy of extrapyramidal symptoms, akathisia, sedation, anticholinergic side-effects (e.g., blurred vision, constipation, dry mouth or hyposalivation and urinary retention), and hyperprolactinaemia that could also be used a weak proxy of sexual side-effects [26, 27].

Forest plots present effect-sizes and their 95% credible intervals of the comparisons between antipsychotics and placebo. Relative risks are used as the effect-size in the forest plots, since they can be more intuitive than other measures such as odds ratios, standardized-mean-differences, or even mean-differences (e.g., in scale scores for overall efficacy and extrapyramidal symptoms or ng/ml for prolactin levels) [28]. However, mean-differences are presented for weight gain (in kg) because of their straightforward understanding for this outcome. When relative risks are not originally reported in the network meta-analysis [1], they are converted from other effect-sizes indices [29] (eAppendix 2). We prefer relative over absolute measures (e.g., absolute risk of a patient to have a side-effect) because they are generally constant across different baseline risks and definitions of the outcome [30], which are common in meta-analysis, especially for the side-effects of antipsychotics [31]. In addition, absolute measures require a baseline risk that can be difficult to be estimated for the individual. Nevertheless, the risk in the placebo group can be presented and discussed, i.e., the weighted percentage of patients in the placebo group with a positive response to treatment or a side-effect.

Patients and their physicians see first the forest plot of efficacy, in which antipsychotics are ranked from the best to the worst efficacy according to the ranking in the network meta-analysis [1]. Then, they can add or remove forest plots for the different side-effects. In that way, they can simultaneously look at effect-sizes of antipsychotics across different outcomes and change the ranking of antipsychotics based on one of these outcomes. They can remove antipsychotics from the list and provide reasons for exclusion. In the end of this step, patients and physicians select one (or more) antipsychotics as potential candidates to be used.

**Fig. 4:**
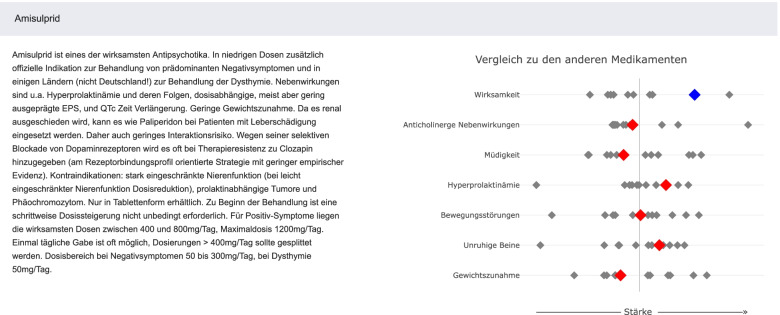
Summary description and summary plot for amisulpride. The short summary for each antipsychotic (e.g., amisulpride) consists of a text description and a plot that presents Z-scores of relative risks across outcomes, which can show the relationship of the relative risk of amisulpride in comparison to the average relative risk of all medications. The figure was taken as a screenshot from the SDM-assistant for choosing antipsychotics

In order to facilitate the process, there are help buttons. In addition, there are short text descriptions for each outcome, and short summaries for each antipsychotic based on the findings of the network meta-analysis [1], treatment guidelines [4], reference textbooks [32] and the respective product leaflets (Fig. 4). The short summaries for each antipsychotic consist of a text description that provides information about the unique indications, contraindications, dosing, pharmacokinetics, and side-effect profile of the selected antipsychotic (also considering side-effects not presented in the forest plots, such as QTc prolongation, myocarditis and agranulocytosis etc.), and a plot that illustrates the relationship of the relative risk of the antipsychotic in comparison to the average relative risk of all antipsychotics with z-scores (eAppendix 2).

In a third step, patients and their physicians look at an overview of patient’s goals and preferences, clinically relevant patient characteristics, selected and excluded antipsychotic medications. They have the chance to further discuss and modify their selections.

In a fourth step, they finalize the antipsychotic selection and save the session.

### Outcomes

Recruitment and data collection will take place at the participating wards. Baseline data will be measured at inclusion and before using the SDM-assistant (T0). Then, the aim is that patients and physicians will use the SDM-assistant at least once during inpatient stay whenever a decision on choosing an antipsychotic is warranted, but no later than three weeks after admission. Both will also be encouraged to use the SDM-assistant as often as possible. At the time of discharge from the hospital, outcomes will be measured (T1). Three months after the discharge, we will contact each patient and the treating outpatient psychiatrist via telephone and/or post in order to obtain follow-up outcome data (T2). Table 1 presents a schematic diagram of participant timeline and data collection. Scales (German versions) could be filled accordingly by patients, their treating physicians or the study personnel (e.g., clinical psychologists not involved in the treatment of the patients and trained to administer the scales). Physicians and study personnel will receive training on the use of individual scales.

**Table 1:**
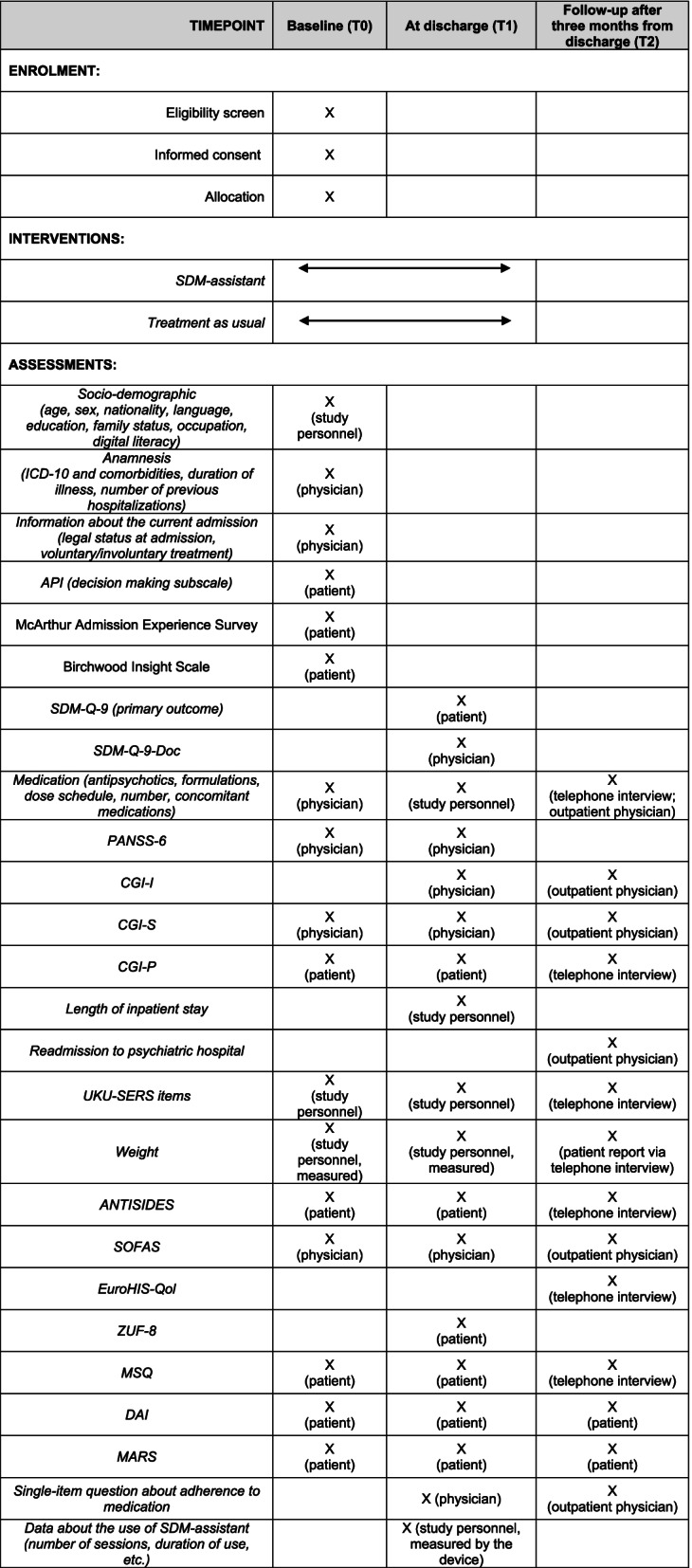
Planned timepoints and data collection

#### Baseline parameters

We will record at baseline (T0) socio-demographics (i.e., age, sex, nationality, language, education, family and occupation status, digital literacy), anamnesis (ICD-10 diagnoses, duration of illness, number of previous hospitalizations, previously prescribed medications), and information about the current admission (legal status, voluntary or involuntary treatment with antipsychotics).

We will also record patient-related factors that could influence the shared-decision making for selecting treatments, i.e. patients’ participation preferences for decision making using the corresponding subscale of the Autonomy Preference Index [33], insight and perceived necessity of treatment using the Birchwood Insight Scale [34] and perception of the current admission using the MacArthur Admission Experience Survey [35].

#### Primary outcome

The primary outcome will be patients’ perceived involvement in decision-making using the Shared-Decision-Making Questionnaire (SDM-Q-9) [36] at T1. The SDM-Q-9 is a self-report scale consisting of nine items that describe different parts of the SDM process and reflecting the inpatient decision-making, e.g., here on the selection of antipsychotic medication. Each item will be scored from 0 “completely disagree” to 5 “completely agree”, and a total score will be calculated ranging from 0 to 45, with a higher score indicated a better-perceived involvement [36]. We expect that the use of the SDM-assistant would substantially improve patients’ perceived involvement, thus identifying a clinically meaningful mean difference of at least 15 points in SDM-Q-9 between the intervention and control group [37, 38].

#### Secondary outcomes and other variables

In addition to the patient’s perceived involvement, we will also investigate physicians’ perceived involvement in decision-making as a secondary outcome using the physician version of the SDM-Q-9 (SDM-Q-Doc) [39] at T1. This will refer to the perceived involvement of the inpatient physician, and any change of the treating physician during the inpatient stay will be recorded.

Apart from the potential improvement in shared-decision-making, we will investigate whether the SDM-assistant could facilitate a more appropriate selection of antipsychotic, as reflected by lower symptom severity and fewer adverse events, higher quality of life and functioning, higher treatment satisfaction and improved adherence.

We will record information about the prescribed antipsychotic medications (e.g., dose schedule, formulation, monotherapy or polypharmacy, concomitant medications) at T0, T1 and T2.

Symptom severity and improvement will be measured at T0 and T1, using the clinician-rated Positive and Negative Syndrome Scale 6-item version (PANSS-6) [40]. PANSS-6 is a recently validated abbreviated version of the original PANSS scale [41], consisting of six items of core schizophrenia symptoms (P1 = delusions, P2 = conceptual disorganization, P3 = hallucinations, N1 = blunted affect, N4 = social withdrawal, N6 = lack of spontaneity/flow of conversation) [40, 42–44]. We will also investigate the clinician-rated Clinical Global Impression-Severity and Improvement (CGI-S, CGI-I) [45], and the patient-rated Clinical Global Impression (CGI-P) [46]. In addition, we will record the length of inpatient stay at T1, and the outpatient psychiatrist will be asked at T2 to document any readmission to the hospital of the patient.

Side-effects will be recorded using items of the clinician version of the Udvalg for kliniske undersøgelser scale (UKU-SERS) [47] at T0, T1 and T2. The UKU scale consists of 48 items covering a comprehensive list of side-effects grouped in four categories, i.e., psychic, neurologic, autonomic and other side-effects. We will also evaluate two additional items about suicidality and oedema. Each item is scored from 0 to 3 based on the severity with a higher score indicating more severity [47]. It will be administered via a semi-structured interview by the study personnel. The weight of patients will be measured at T0 and T1, and we will ask the patients to report their weight at T2. Furthermore, we developed the patient-reported antipsychotic and antidepressant side-effect scale (ANTISIDES) that will be administered at T0, T1 and T2. The scale consists of 41 items covering a broad spectrum of side effects that can be caused by psychotropic drugs. Each item will be rated on a visual analogue scale from 0 “not at all” to 10 “extremely strong”, supplemented by an assessment of the impairment experienced due to the respective side effect (eAppendix 3).

We will address global functioning using the clinician-rated Social and Occupational Functioning Assessment Scale (SOFAS) [48] at T0, T1 and T2, and quality of life using the patient-reported EUROHIS-QOL [49] at T2.

Treatment satisfaction, attitudes and adherence will be measured using the patient-reported scales Questionnaire on Patients’ Treatment Satisfaction (ZUF8) [39], Medication Satisfaction Questionnaire (MSQ) [50], Drug Attitude Inventory (DAI) [51] and Medication Adherence Rating Scale (MARS) [52] at T0, T1 and T2. Physicians will also rate adherence to the medication treatment at T1 and T2 using a single question scored on a 4-point Likert scale from 1 “very good” to 4 “poor”.

Last, data about the use of the SDM-assistant (e.g., number of sessions, duration of use) will be recorded within the SDM-assistant and evaluated at T1. This information would also be helpful in assessing treatment integrity.

#### Qualitative data

We will also obtain qualitative data in order to gain insights into the treatment integrity and mode of action of using the SDM-assistant for choosing antipsychotics, as well as identify further potential influencing factors. The combination of quantitative (hypothesis-driven) and qualitative approach would increase the validity and interpretation of the findings of the trial. For this purpose, we will use purposive sampling in order to identify individual cases of successful/unsuccessful joint decisions or antipsychotic selection. We will conduct interviews with about 15 patients from the intervention group until no fundamentally new aspects will be added. Possible inclusion criteria are as follows: 1) patient or physician are dissatisfied with the use of SDM-assistant and/or do not want to use it in the future, 2) patient or physician discontinue the use of SDM-assistant, 3) patient or physician disagree with the outcome of the SDM-assistant. 4) patient or physician satisfaction with the SDM-assistant is far above average, 5) the use or the outcome of the SDM-assistant triggers unusual or unexpected reactions in the patient or physician. Physicians will be interviewed separately and in terms of their experience across several uses of the app with their patients. The interviews will be guideline-based and conducted by the study personnel, recorded acoustically, then transcribed and qualitatively analysed using content analysis [53].

### Sample size and recruitment

The target sample size is *N* = 252 patients from *n* = 14 clusters (126 participants from 7 intervention wards and 126 participants from 7 control wards). Power calculation estimated *N* = 180 participants (*n* = 12 clusters) to be analysed in order to detect an expected Cohen’s d of 0.45–0.5 in the primary outcome of SDM-Q-9 with a power of at least 80% at two-sided alpha of 0.05 assuming ICC = 0.02. These assumptions are based on data from a comparable study (SDM-PLUS), which involved a similar population and the same main outcome parameter [38]. Due to an assumed drop-out rate of patients of 15–25% and to account for a potential drop out of clusters, we aim at recruiting *n* = 14 clusters and *N* = 252 patients.

Patient recruitment will be conducted by a trained study staff and will take place on the 14 selected wards at three hospitals in Munich, Germany (Klinikum rechts der Isar of the Technical University of Munich, kbo-Isar-Amper-Klinikum München-Ost and München-Schwabing). These hospitals with more than 1500 beds in total can facilitate the enrolment of the planned number of patients (*N* = 252) in a timely manner, as in our previous trials [38, 54].

### Randomization and blinding

Randomization will take place at the cluster (i.e., ward) level, in order to minimize contamination effects inherent to the nature of the intervention in case the randomization was performed at the patient or physician level [55]. The investigators will determine seven pairs of comparable wards (regarding the treatment spectrum, the size of the ward, etc.) and then randomize one ward of each pair either to the intervention or to the control group. While the principal investigators will determine the paired wards, the randomization will be conducted by a blinded investigator independent to the study using the coin toss method. However, due to the nature of the intervention (use of the SDM-assistant vs. TAU), there will be no blinding between intervention and control wards.

### Data analysis

The effect-size for continuous outcomes will be the mean difference (MD) and for dichotomous outcomes will be the odds ratio (OR), presented with their 95% confidence intervals. Per-protocol analysis will be conducted, i.e., by excluding patients with no outcome data, patients that do not receive at least one session of SDM-assistant, patients who moved from a ward belonging to the control group to a ward belonging to the intervention group or vice versa. There will be no imputation of missing outcome data, and observed cases will be used.

The primary outcome (SDM-Q-9 at T1) and secondary continuous outcomes will be analysed with mixed-effects linear regression models fitted with ward (cluster) as a random-effects term and with intervention/control group as a fixed-effects term. In a similar vein, dichotomous outcomes will be analysed with logistic regression models using the generalised estimating equations (GEEs).

The robustness of the results of the primary outcome will be investigated in a sensitivity analysis by adjusting for baseline covariates such as severity of illness, admission status and variables with a large baseline imbalance.

In addition, we plan exploratory subgroup analyses in order to investigate if there is potential effect-modification of patient characteristics (e.g., age, sex, symptom severity, duration of illness) on the primary outcome.

Alpha will be set at two-sided alpha of 0.05.

## Discussion

Choosing an antipsychotic medication is an important medical decision that should require benefit-risk assessments and the active involvement of patients whenever possible (i.e., shared-decision making) [4, 5]. In order to facilitate shared-decision making for choosing antipsychotics, we developed an evidence-based shared-decision making assistant (SDM-assistant). In the planned cluster-randomised trial, we will examine the use of SDM-assistant for choosing antipsychotics in hospitalized patients with an acute exacerbation of schizophrenia. We will evaluate the perceived involvement of patients in the decision-making as primary outcome, and therapeutic alliance, symptom severity, side-effects, treatment satisfaction, adherence, quality of life, functioning and rehospitalizations as secondary outcomes.

There are potential methodological limitations and challenges.

First, the SDM-assistant aims to present high-quality evidence from a network meta-analysis on the efficacy and side-effects of antipsychotics [1]. However, not every important side-effect is covered, for example cognitive and sexual side-effects (yet prolactin elevation used as a proxy of sexual side-effects, as discussed above) [56, 57], since they have not been well examined in network meta-analyses. In addition, the current version of SDM-assistant is focusing on the selection of antipsychotics in the acute phase, and other aspects of antipsychotic treatment are not considered, long-term treatment, management of adverse events, switching antipsychotics, dosing etc. Moreover, we focused on medication issues, yet we acknowledge that the treatment of schizophrenia is multimodal, e.g., psychological treatments have also a major role [58, 59]. In future updates, SDM-assistant could implement new data from network meta-analysis (or other sources of high-quality evidence), expand the list of side-effects and antipsychotics, as well as consider additional treatment modalities (e.g., psychological treatments and non-invasive brain stimulation) and extend the framework to facilitate a broader range of decisions. Additional features could also be added such as giving the option to select different effect-size indices (e.g., absolute percentages) [29] or different plots for presentation (e.g., Cates or Kilim plots, etc.) [60, 61]. Furthermore, recent meta-analytic methods that have been developed to individualise treatment recommendations, e.g., [62, 63] were not yet implemented. The use of such complicated methods in shared-decision making requires caution, since they could swift decision-making to a computerised paternalistic process that is heavily dependent on statistical assumptions [16].

Third, there are certain difficulties in the implementation of the intervention. Shared-decision making can be successful, yet challenging, in acutely ill inpatients with schizophrenia, potentially due to reduced decisional capacity, high severity psychotic symptoms, cognitive impairment, lack of insight, and negative attitudes towards the treatment [64–66]. The use of the SDM-assistant could make the process even more demanding due to the higher attention and cooperation requirements. For these reasons, we will use a mixed-methods approach to evaluate barriers and facilitators for the implementation of the intervention. SDM-assistant aims to enable patients to get familiar with the evidence and participate in the decision-making, without forcing them to be involved. In those cases, a more paternalistic decision-making approach with the physician choosing a suitable medication might be still acceptable [18]. Nevertheless, the early involvement of patients in the selection of antipsychotics can be very important in order to prevent burdensome side-effects, since at least some of them could be subjectively realized only later after the improvement of symptoms, e.g. rapid weight gain [67]. Third, treatment-as-usual will be used in the control group, instead of an active control group (e.g., using paper forms to present evidence on antipsychotics or unspecific group training) in order to avoid contamination. However, it is possible that the increased attention in the intervention group could inflate any differences found.

In conclusion, we developed SDM-assistant, an SDM assistant that presents high-quality evidence on the efficacy and side-effect profile of antipsychotics and can be used as a framework for shared-decision making between physicians and patients with schizophrenia when selecting antipsychotic medications. The major aim of the planned trial is to show that the use of SDM-assistant leads to increased patients’ perceived involvement in the decision-making process as well as to a more suitable selection of antipsychotics, as reflected by our secondary outcomes (e.g., symptom severity, side-effects, adherence, treatment satisfaction, rehospitalizations). The implementation of this intervention is challenging in acutely ill patients with schizophrenia, and potential barrier and facilitators will be examined. Last, according to one of the principles of evidence-based medicine [68], we hope that SDM-assistant can successfully advance evidence-based medicine in schizophrenia by putting medical evidence on antipsychotics into the context of patient preferences and values.

## Supplementary Information

**Additional file 1.** eAppendix.

## Data Availability

Not applicable.

## Abbreviations

ANTISIDES: Antipsychotic and antidepressant side-effect scale
API: Autonomy Preference Index
BIS: Birchwood Insight Scale
CGI-I: Clinical Global Impression Improvement scale
CGI-P: Clinical Global Impression Patient scale
CGI-S: Clinical Global Impression Severity scale
CRF: Case Report Form
DAI: Drug Attitude Inventory
GEE: Generalised estimating equations
ICD: International Classification of Diseases
IPDAS: International Patient Decision Aids Standards instrument
kg: kilogram
MAES: MacArthur Admission Experience Survey
MARS: Medication Adherence Rating Scale
MD: Mean difference
MSQ: Medication Satisfaction Questionnaire
OR: Odds ratio
PANSS-6: Positive and Negative Syndrome Scale
SDM: Shared-Decision-Making
SDM-assistant: Shared-Decision Making Assistant for choosing antipsychotics
SDM-Q-9: Shared Decision Making Questionnaire 9-tem patient version
SDM-Q-9-Doc: Shared-Decision Making Questionnaire 9-item physician version
SOFAS: Social and Occupational Functioning Assessment Scale
TAU: Treatment-As-Usual
UKU-SERS: Udvalg for kliniske undersøgelser Side Effect Rating scale
