# Supplementary material for: Evidence-based Shared-Decision-Making Assistant (SDM-assistant) for choosing antipsychotics: protocol of a cluster-randomized trial in hospitalized patients with schizophrenia
Source: BMC Psychiatry. 2022 Jun 17;22:406. doi: 10.1186/s12888-022-04036-5 (PMC9204887; doi:10.1186/s12888-022-04036-5)
Supplement: Supplementary file 1 — Additional file 1. eAppendix. [file 12888_2022_4036_MOESM1_ESM.docx]

**eAppendix**

**Evidence-based shared-decision-making assistant (SDM-assistant) for choosing antipsychotics: protocol of a cluster-randomized trials in inpatients with schizophrenia**

Spyridon Siafis,^1^ Nicola Bursch,^1^ Katharina Müller,^2^ Lisa Schmid,^1^ Florian Schuster,^3^ Jakob Waibel,^4^ Tri Huynh,^4^ Florian Matthes,^4^ Alessandro Rodolico,^5^ Peter Brieger,^2^ Markus Bühner,^6^ Stephan Heres,^2^ Stefan Leucht,^1*^ Johannes Hamann^1*^

^1^Department of Psychiatry and Psychotherapy, School of Medicine, Technical University of Munich, Munich, Germany

^2^kbo-Isar-Amper-Klinik, Munich, Germany

^3^Schön Klinik Roseneck, Rosenheim, Germany

^4^Department of Informatics, Technical University of Munich, Munich, Germany

^5^Department of Clinical and Experimental Medicine, Institute of Psychiatry, University of Catania, Catania, Italy.

^6^Psychological Methodology and Diagnostics, Ludwig Maximilian University, Munich, Germany

*Contributed equally

Table of Contents

[1. IPDAS criteria 3](#_Toc104192013)

[2. Converting effect-sizes 11](#_Toc104192014)

[3. Antipsychotic and antidepressant side-effect scale (ANTISIDES): Stefan Leucht, Claudia Leucht, Spyridon Siafis, Elfriede Scheuring, Wulf-Peter Hansen, Alessandro Rodolico, Katharina Müller, Nicola Bursch, Lisa Schmid, Johannes Hamann 12](#_Toc104192015)

[4. References 16](#_Toc104192016)

# 1. IPDAS criteria

The SDM-assistant fulfilled the relevant qualifying and certifying criteria of IPDASi v4.0 (1), and considered many of the relevant quality criteria.

| **IPDAS dimensions** | **Qualifying criteria** | | **Certifying criteria** | | **Quality criteria** | |
| --- | --- | --- | --- | --- | --- | --- |
|  | Item | Comment | Item | Comment | Item | Comment |
| **Information** | *1. The patient decision aid describes the health condition or problem (treatment, procedure, or investigation) for which the index decision is required. (4.84)* | Yes, the health condition or problem is clearly stated (i.e., the large variety of different antipsychotics for the treatment of schizophrenia) | *1. The patient decision aid shows the negative and positive features of options with equal detail (e.g., using similar fonts, sequence, presentation of statistical information). (4.54)* | Yes, positive and negative features (i.e., more efficacy and less side-effects vs. less efficacy and more side-effects) are presented in the same way in plots and using the same effect-size. | *1. The patient decision aid describes the natural course of the health condition or problem, if no action is taken (when appropriate). (4.44)* | Yes, if no action is taken, then antipsychotic selection is done within the standard-care (e.g., the physician chooses an antipsychotic). A short description of this paternalistic model and shared-decision making is provided. In addition, links to further information are provided for not using antipsychotics. |
|  | *2. The patient decision aid explicitly states the decision that needs to be considered (index decision). (4.66)* | Yes, the decision to be considered is clearly stated (i.e., selection of antipsychotic medication) |  | | *2. The patient decision aid makes it possible to compare the positive and negative features of the available options. (4.21)* | Yes, there are plots to compare the positive and negative features of the available options (different drugs). |
|  | *3. The patient decision aid describes the options available for the index decision. (4.99)* | Yes, the relevant antipsychotics used in clinical practice are described. |  |  |  | |
|  | *4. The patient decision aid describes the positive features (benefits or advantages) of each option. (4.80)* | Yes, the positive features are described, i.e., more efficacy and less side-effects |  |  |  |  |
|  | *5. The patient decision aid describes the negative features (harms, side effects, or disadvantages) of each option. (4.89)* | Yes, the negative features are described, i.e., more side-effects and less efficacy |  |  |  |  |
| **Probabilities** |  | |  | | *3. The patient decision aid provides information about outcome probabilities associated with the options (i.e., the likely consequences of decisions). (4.52)* | Yes, outcomes are presented with relative risks or mean differences for weight gain. |
|  |  |  |  |  | *4. The patient decision aid specifies the defined group (reference class) of patients for whom the outcome probabilities apply. (4.33)* | Yes, it is described that effect-sizes of the comparisons of antipsychotics with placebo in patients with an acute exacerbation of schizophrenia are provided. |
|  |  |  |  |  | *5. The patient decision aid specifies the event rates for the outcome probabilities (4.23)* | Relative measures are presented in the forest plots, since they are more stable in comparison to absolute measures. It is also not easy to estimate the baseline risk for the individual patient. |
|  |  |  |  |  | *6. The patient decision aid allows the user to compare outcome probabilities across options using the same time period (when feasible).*  *(3.84) Amended to include comparison, and ‘when feasible’’ added.* | Yes, all outcomes are presented at about six weeks of treatment with an antipsychotic. Effect-sizes of multiple antipsychotics and outcomes are presented simultaneously. |
|  |  |  |  |  | *7. The patient decision aid allows the user to compare outcome probabilities across options using the same denominator (when feasible). (4.46)* | Yes, relative measures are used, using placebo as reference. |
|  |  |  |  |  | *8. The patient decision aid provides more than 1 way of viewing the probabilities (e.g., words, numbers, and diagrams). (3.43)* | Yes, there is a text description for each drug and two different types of graphs. |
| **Values** | 6. The patient decision aid describes what it is like to experience the consequences of the options (e.g., physical, psychological, social) | Yes, the consequences of the options are given as efficacy and side-effects, and short descriptions of them are provided. |  | | *9. The patient decision aid asks patients to think about which positive and negative features of the options matter most to them (implicitly or explicitly).* | Yes, the patient thinks and notes which side-effects are the most important. The SDM-assistant provides a framework of the discussion of these preferences. |
| **Guidance** |  | |  | | *10. The patient decision aid provides a step-by step way to make a decision. (2.97)* | Yes, the SDM-assistant provides a multi-step framework for shared-decision making in the selection of antipsychotic medication. |
|  |  |  |  |  | *11. The patient decision aid includes tools like worksheets or lists of questions to use when discussing options with a practitioner. (3.17)* | The forest plots and summaries would provide the framework of the discussion of the options with the practitioner. |
| **Development** |  | |  | | *12. The development process included a needs assessment with clients or patients. (4.51)* | Yes, ex-users were included in the development and design of the tool. |
|  |  |  |  |  | *13. The development process included a needs assessment with health professionals. (4.07)* | Yes, psychiatrists were included in the development and design of the tool. |
|  |  |  |  |  | *14. The development process included review by clients/patients not involved in producing the decision support intervention. (3/71)* | In focus groups with patients. |
|  |  |  |  |  | *15. Τhe development process included review by professionals not involved in producing the decision support intervention. (3.54)* | In focus groups with psychiatrists. |
|  |  |  |  |  | *16. The patient decision aid was field tested with patients who were facing the decision. (4.56)* | Yes, the tool has been pre-tested in patients who were facing the decision. This evaluation will also be part of this trial. |
|  |  |  |  |  | *17. The patient decision aid was field tested with practitioners who counsel patients who face the decision. (4.56)* | Yes, the tool has been pre-tested by practitioners who counsel patients who face the decision. This evaluation will also be part of this trial. |
| **Evidence** | ﻿ | | *2. The patient decision aid (or associated documentation) provides citations to the evidence selected. (3.85) ‘‘Studies’’ changed tο ‘‘evidence.’’* | Yes, citations are provided from the sources of evidence: NMA (2, 3), national guidelines (4), and reference textbooks (5). | *18. The patient decision aid (or associated documentation) describes how research evidence was selected or synthesized. (3.49)* | Yes, the results of NMA were used, since they provide high quality evidence with a clear impact in clinical practice. This is stated in the videos of the tool. |
|  |  |  | *3. The patient decision aid (or associated documentation) provides a production or publication date. (4.02)* | Yes, the production date and version are provided in the tool. | *19. The patient decision aid (or associated documentation) describes the quality of the research evidence used. (3.77)* | Yes, the results of NMA were used, since they provide high quality evidence with a clear impact in clinical practice. This is stated in the videos of the tool. Limitations of the evidence are also noted. |
|  |  |  | *4. The patient decision aid (or associated documentation) provides information about the update policy. (2.99)* | Yes, the update policy is provided. For the clinical trial, no major update is planned and any change or update will be mentioned and noted in the version. |  | |
|  |  |  | *5. The patient decision aid provides information about the levels of uncertainty around event or outcome probabilities (e.g., by giving a range or by using phases such as ‘‘our best estimate is*  *. . .’’). (3.53)* | Yes, the average effect-size is presented along with the credible intervals in the main forest plots. |  |  |
| **Disclosure** |  | | *6. The patient decision aid (or associated documentation) provides information about the funding source used for development. (4.39)* | Yes, the funding source is provided (see the manuscript too). | *20. The patient decision aid includes authors’/ developers’ credentials or qualifications. (3.51)* | Yes |
| **Plain language** |  | |  | | *21. The patient decision aid (or associated documentation) reports readability levels (using 1 or more of the available scales). (3.06)* | Readability levels are not provided. However, the test was pre-tested with practitioners and patients. |
| **Evaluation** |  | |  |  | *22. There is evidence that the patient decision aid improves the match between the preferences of the informed patient and the option that is chosen. (3.44)* | This would be tested in the planned clinical trial. |
|  |  |  |  | | *23. There is evidence that the patient decision aid helps patients improve their knowledge about options’ features. (3.67)* | This would be tested in the planned clinical trial. |
| **Test** |  | | *7. The patient decision aid describes what the test is designed to measure. (4.90)* | This item is not applicable, since SDM-assistant does not provide a screening or similar test. | *24. The patient decision aid includes information about the chances of having a true-positive test result.(4.74)* | This item is not applicable, since SDM-assistant does not provide a screening or similar test. |
|  |  |  | *8. If the test detects the condition or problem, the patient decision aid describes the next steps typically taken. (4.67)* | This item is not applicable, since SDM-assistant does not provide a screening or similar test. | *25. The patient decision aid includes information about the chances of having a true-negative test result. (4.73)* | This item is not applicable, since SDM-assistant does not provide a screening or similar test. |
|  |  |  | *9. The patient decision aid describes the next steps if the condition or problem is not detected. (4.28)* | This item is not applicable, since SDM-assistant does not provide a screening or similar test. | *26. The patient decision aid includes information about the chances of having a false-positive test result. (4.77)* | This item is not applicable, since SDM-assistant does not provide a screening or similar test.. |
|  |  |  | *10. The patient decision aid has information about the consequences of detecting the condition or disease that would never have caused problems if screening had not been done (lead time bias). (4.56)* | This item is not applicable, since SDM-assistant does not provide a screening or similar test. | *27. The patient decision aid includes information about the chances of having a false-negative test result. (4.78)* | This item is not applicable, since SDM-assistant does not provide a screening or similar test. |
|  |  |  |  | | *28. The patient decision aid describes the chances the disease is detected with and without the use of the test. (4.52)* | This item is not applicable, since SDM-assistant does not provide a screening or similar test. |

# 2. Converting effect-sizes

In the forest plots of the SDM-assistant, relative risks (RR) are presented along with their 95% credible intervals for all outcomes, except for mean difference (MD in kg) for weight gain. These effect-sizes were extracted from a large network meta-analysis on the comparative efficacy and safety of antipsychotics for acute exacerbations in patients with schizophrenia (2). This meta-analysis included short-term trials with a follow-up period of 3-13 weeks. Since there were no shorter-term data on weight gain for perphenazine, we used mean differences from a network meta-analysis on the longer-term metabolic side-effects of antipsychotics (not yet published) (3).

The NMA of Huhn et al 2019 (2) reported relative risks for the number of patients with akathisia, use of antiparkinsonian medication and sedation, as well as mean differences in kg for weight gain. However, it reported standardized mean differences (SMD) for overall efficacy and mean difference in ng/ml for prolactin elevation. Therefore, the latter effect-sizes were converted into relative risks using the formulas of the Cochrane Handbook (6), as it was previously applied in our previous publication (7): 1) from MD to SMD assuming a pooled standard deviation (SD-pooled), 2) from SMD to odds ratios (OR) assuming that the continuous variable follows a logistic distribution with an equal standard deviation between the two groups, 3) from OR to RR assuming an assumed control risk (ACR).

We converted MD for prolactin elevation (in ng/ml) to SMD, using as SD-pooled the weighted average standard deviation of prolactin levels in the dataset of Huhn et al 2020: $SMD=\frac{MD}{SDpooled}$

We converted SMD for prolactin elevation and overall efficacy to OR: $OR= e^{1.81*SMD}$

We converted OR to RR assuming as ACR the weighted average risk in the placebo group: $RR=\frac{OR}{(1-ACR*\left( 1-OR \right))}$.

These formulas were applied for both the point estimate and the 95% credible intervals.

The same procedure was also used to calculate relative risks for weight gain for the summary plots

In the summary plot, the Z-scores of log relative risks were calculated for each outcome and drug, e.g. for drug A: $z(drug A)= \frac{logRR\left( drug A \right)-average\log RR of all drugs}{SD\log RR of all drugs}$.

# 3. Antipsychotic and antidepressant side-effect scale (ANTISIDES)

Authors: Stefan Leucht^1^, Claudia Leucht^2^, Spyridon Siafis^1^, Elfriede Scheuring^3^, Wulf-Peter Hansen^3^, Alessandro Rodolico, Katharina Müller^4^, Nicola Bursch^1^, Lisa Schmid^1^, Johannes Hamann^1^

^1^Department of Psychiatry and Psychotherapy, School of Medicine, Technical University of Munich, Munich, Germany; ^2^Max Planck Institute of Psychiatry, Munich, Germany; ^3^Bündnis gegen Stigma (BASTA), Munich, Germany; ^4^kbo-Isar-Amper-Klinik, Munich, Germany


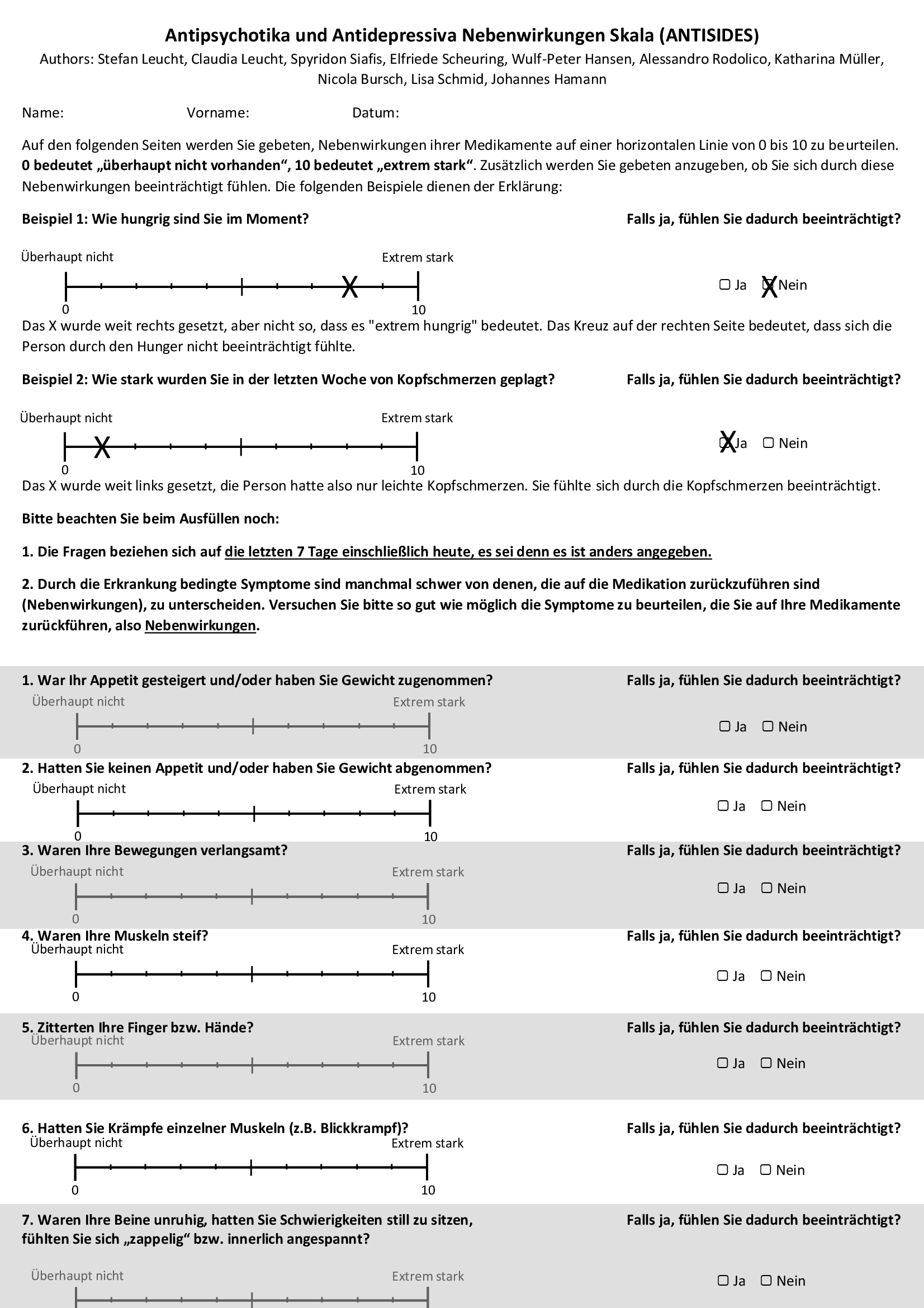


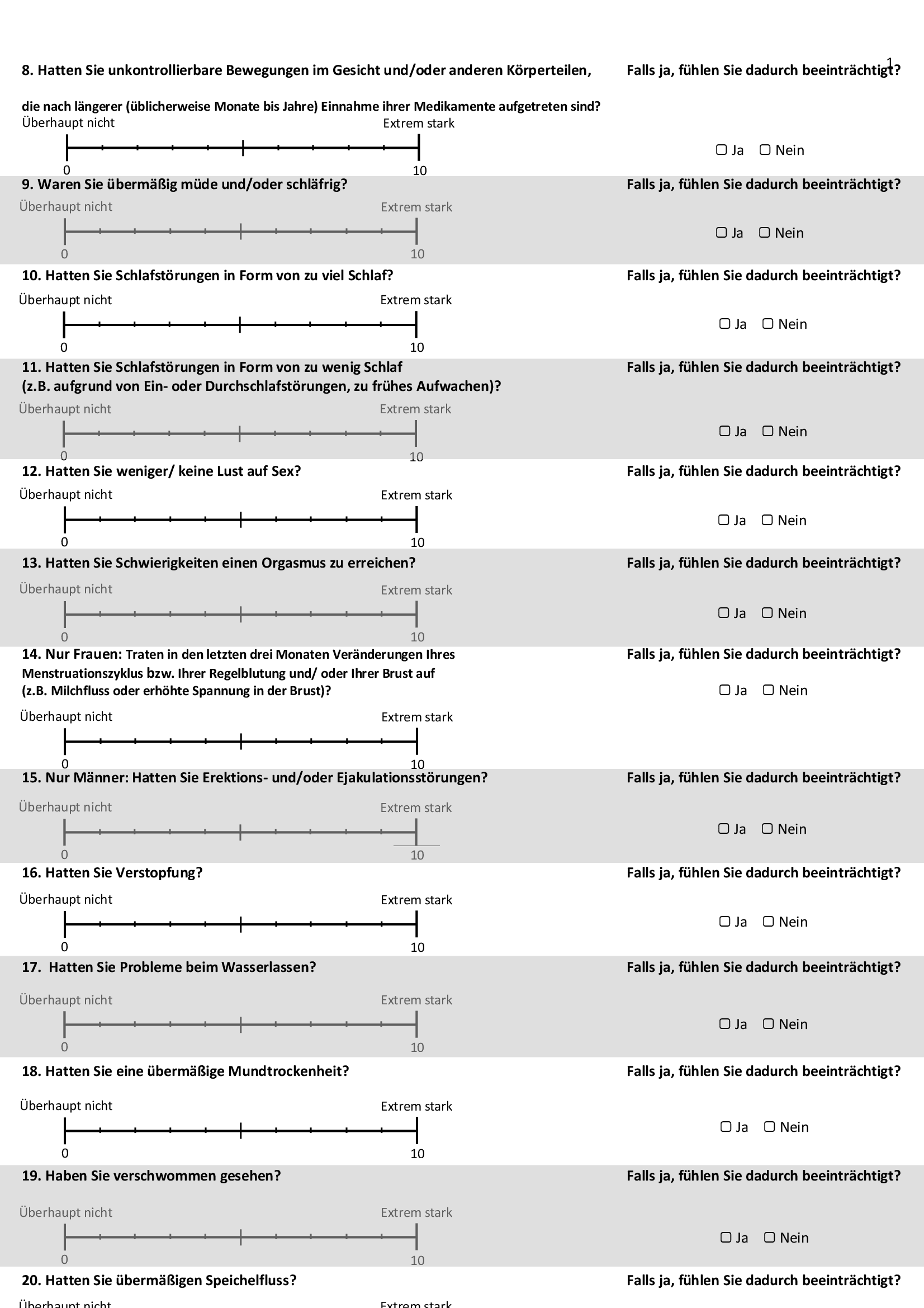


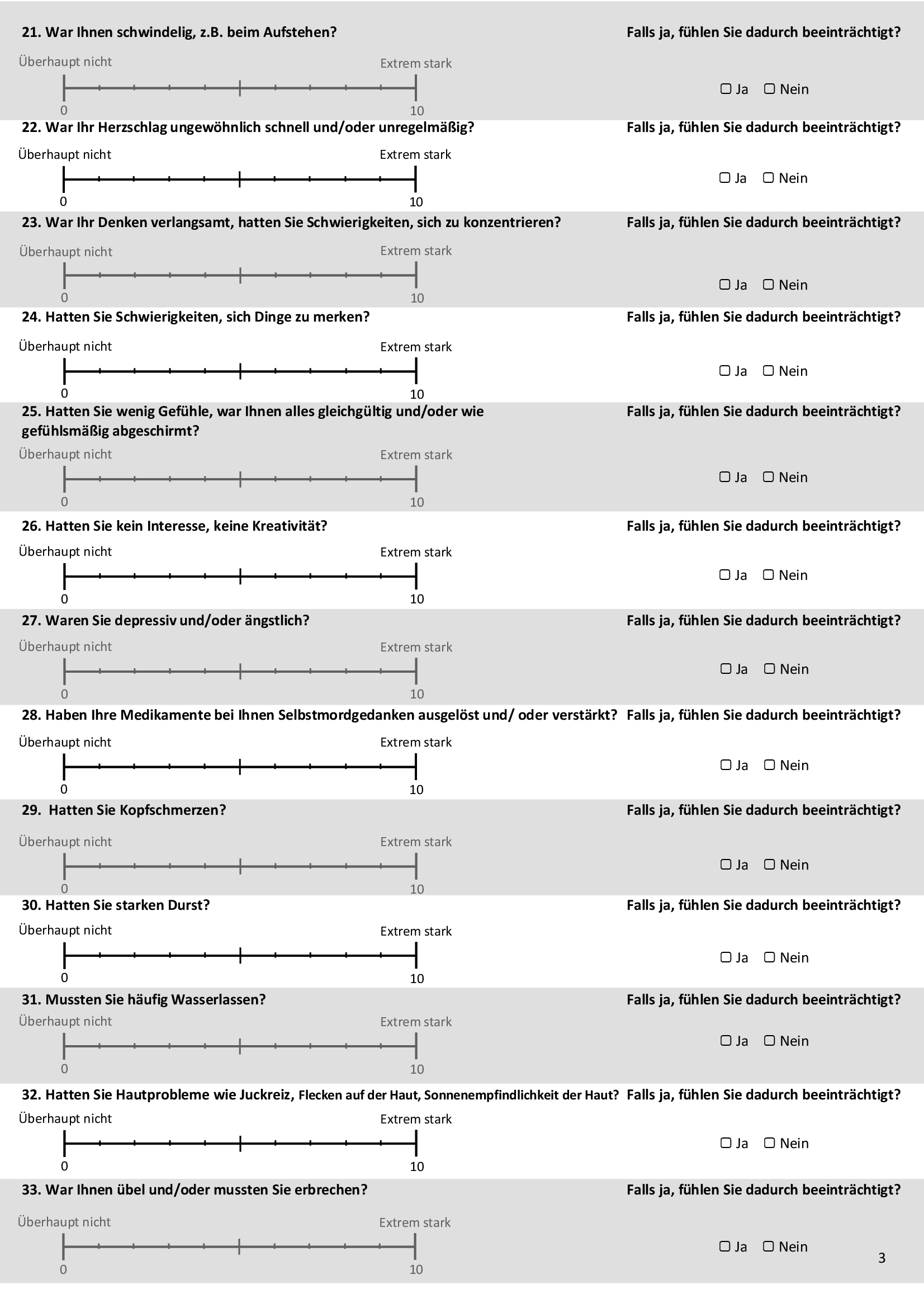


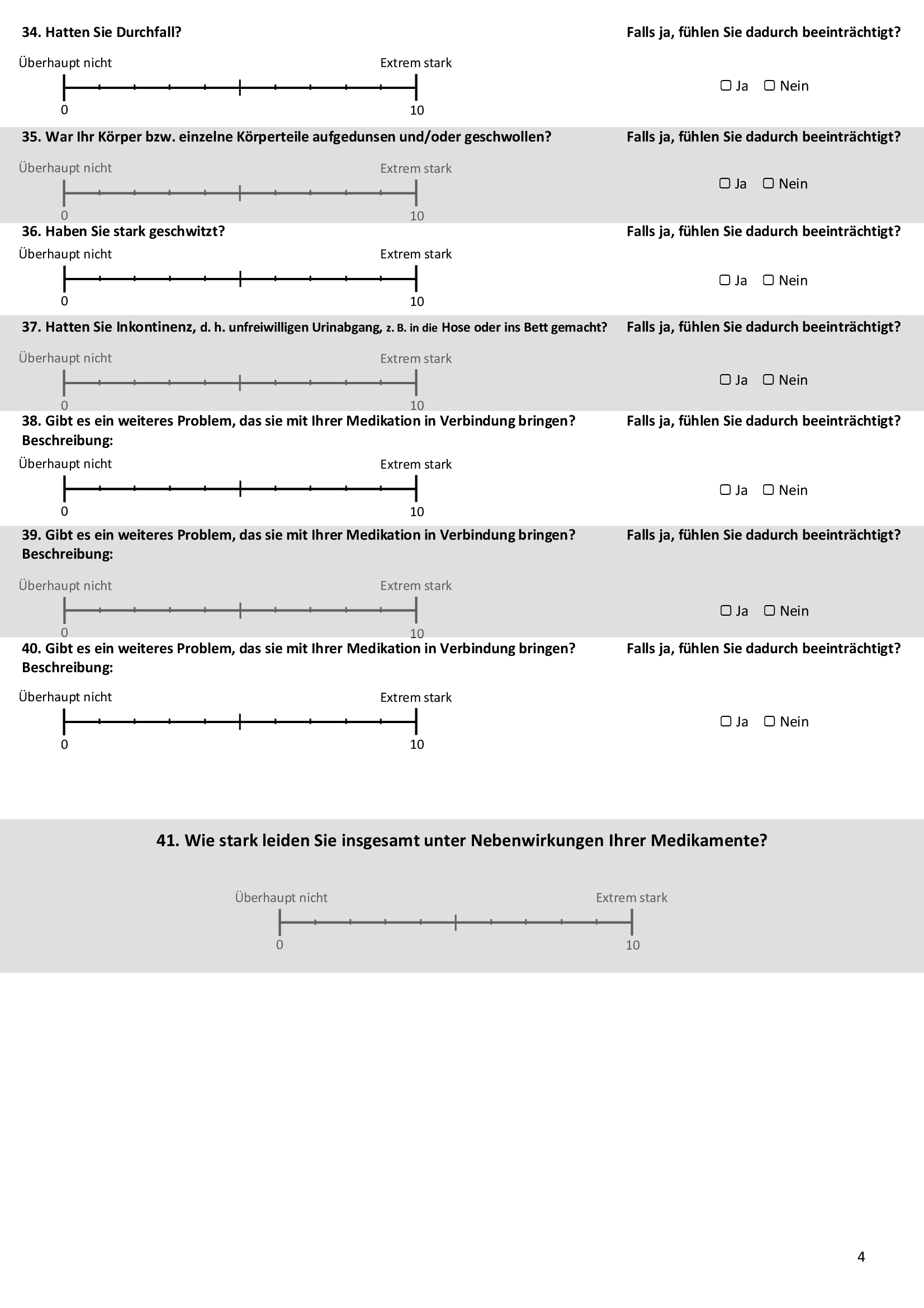


# 4. References

1. Joseph-Williams N, Newcombe R, Politi M, Durand MA, Sivell S, Stacey D, et al. Toward Minimum Standards for Certifying Patient Decision Aids: A Modified Delphi Consensus Process. Med Decis Making. 2014;34(6):699-710.

2. Huhn M, Nikolakopoulou A, Schneider-Thoma J, Krause M, Samara M, Peter N, et al. Comparative efficacy and tolerability of 32 oral antipsychotics for the acute treatment of adults with multi-episode schizophrenia: a systematic review and network meta-analysis. The Lancet. 2019;394(10202):939-51.

3. Schneider-Thoma J, Kapfhammer A, Wang D, Bighelli I, Siafis S, Wu H, et al. Metabolic side effects of antipsychotic drugs in individuals with schizophrenia during medium- to long-term treatment: protocol for a systematic review and network meta-analysis of randomized controlled trials. Syst Rev. 2021;10(1):214.

4. Gaebel W, Hasan A, Falkai P, editors. S3-Leitlinie Schizophrenie. Springer-Verlag; 2019 Oct 29.

5. Benkert O, Hippius H. Kompendium der Psychiatrischen Pharmakotherapie: Springer 2021.

6. Higgins JPT, Thomas J, Chandler J, Cumpston M, Li T, Page MJ, et al. Cochrane handbook for systematic reviews of interventions: John Wiley & Sons; 2019.

7. Leucht S, Siafis S, Engel RR, Schneider-Thoma J, Bighelli I, Cipriani A, et al. How Efficacious Are Antipsychotic Drugs for Schizophrenia? An Interpretation Based on 13 Effect Size Indices. Schizophr Bull. 2021.
